# Effects of Diet and Metformin on placental morphology in Gestational Diabetes Mellitus

**DOI:** 10.12669/pjms.326.10872

**Published:** 2016

**Authors:** Rabia Arshad, Muhammad Adnan Kanpurwala, Nasim Karim, Jahan Ara Hassan

**Affiliations:** 1 Dr. Rabia Arshad, MBBS, M. Phil. Assistant Professor and Head of Pharmacology Department, Altamash Institute of Dental Medicine, 2-R Sunset Boulevard, DHA, Karachi, Pakistan; 2 Dr. Muhammad Adnan Kanpurwala, MBBS, M. Phil. Associate Professor, Physiology Department, Karachi Institute of Medical Sciences, Karachi, Pakistan; 3 Dr. Nasim Karim, MBBS, M. Phil, Ph D, Post Doc. Professor and Head of Pharmacology Department, Bahria University Medical and Dental College, Karachi, Pakistan; 4 Dr. Jahan Ara Hasan, MBBS, FCPS, MCPS. Associate Professor, Gynecology and Obstetrics Department, Dow University of Health Sciences, Karachi, Pakistan

**Keywords:** Gestational diabetes, Diet, Exercise, Metformin, Placenta, Gross morphology, Microscopic morphology

## Abstract

**Objective:**

To evaluate the effects of diet control and Metformin on placental morphology in gestational diabetes mellitus (GDM).

**Methods:**

After written informed consent 62 GDMs were enrolled. According to WHO criteria, 30 cases of GDMs with blood sugar level <130 mg/dl, were assigned Group B (2000-2500Kcal/day and 30 minute walk thrice weekly were kept on diet control and 32 cases of GDM with blood sugar level >130 mg/dl, assigned Group C were kept on diet with tablet Metformin,(500mg TDS) Finally 25 normal pregnant females were kept in Group A as control. After delivery placentae were preserved and evaluated for morphology.

**Results:**

Heavy placentae with abundant villous immaturity, chorangiosis and syncytial knots in group B and fibrinoid necrosis and calcification in group C were seen. In group B versus A placental and cord width while in Group C versus A only cord width in gross morphology showed significant results. In group B versus A villous immaturity, chorangiosis, infarction and syncytial knots in light microscopy were present; similarly in B versus C placental width, chorangiosis and syncytial knots showed significant results, while in C versus A results were non-significant.

**Conclusion:**

Metformin produced beneficial effects on placental morphology being comparable to normal control in contrast to diet group.

## INTRODUCTION

Gestational diabetes is glucose intolerance identified in the second trimester of pregnancy. This occurs mainly due to the diabetogenic effects of placental hormones and is associated with certain critical fetal and maternal consequences.^1^ The worldwide prevalence of GDM is 3-9% whereas in our population prevalence is 3-3.45% but the outcomes are much worse due to lack of knowledge and medical care facilities.^2^

Placenta is involved in the nutritional supply of the maturing fetus. It is a discoid shaped structure covered with membranes and microscopically composed of multiple villi containing minute blood vessels and mesenchymal supportive tissue. Change in the maternal atmosphere influences the physiology and structure of this central organ.^3^ The placenta exhibits multiple gross and microscopic alterations in gestational diabetes mellitus such as increase in the proliferative rate of trophoblasts, stromal cells and villous capillaries of placental tissue. This mainly happens due to enhanced effects of endogenous fetal insulin. Other main feature is hypoxia, which occurs in the placental tissue due to increase in fetal size (macrosomia) and demands. This subsequently leads to increase in the weight, diameter and thickness of placenta.^4^ Excessive amount of glucose in maternal blood also gets stored in the placental tissue.^5^ Patho-physiology can be linked to altered levels of placental vascular endothelial growth factor, insulin and other related growth factors (IGF1, IGF2 and IGF binding proteins) which regulate the placental development and growth.^6^ This results ultimately in increase in size and weight of the placenta. Furthermore on microscopic evaluation, all hypoxic parameters are notably increased in placentae of GDM patients.^7^

Diet and exercise is the necessary management but patients with higher glucose level also need pharmacological management. Insulin is a gold standard therapy but it was seen that even with insulin therapy there were chances of maternal weight gain, macrosomic babies and unexplained term deaths of fetus and still births. There are well documented hypoxic changes in placenta due to use of insulin.^8^ In contradiction to the past when oral anti-diabetic drugs were thought to be teratogenic,^8^ in the recent years, Metformin is said to be the safest as classified category B drug. It produces euglycemia, by improving insulin resistance, enhances glucose uptake and also decreases intracellular glucose production. Furthermore, it improves capillary functions, reduces hyperglycemia, and decreases the onset and severity of micro and macro vascular complications in type 2 diabetes.^9^ Therapeutic trials have also confirmed that during pregnancy Metformin has good patient compliance, with no significant side effects.^10^ On account of paucity of data regarding effects of metformin on placental morphology, present study was designed to compare the effect of diet control with Metformin on placental morphology in gestational diabetes mellitus (GDM).

## METHODS

This study was conducted at Lyari General Hospital, Civil Hospital and Mamji Hospital Karachi from June 2010- June 2011. For this clinical trial study, fasting and random blood sugar level of the patients attending antenatal clinic were checked. Diagnosis of GDM according to WHO criteria (FBS > 100mg/dl and RBS > 126mg/dl) was made using glucometer and rechecked with lab reports. In high risk patients such as with previous GDM or bad obstetrical history, diagnosis was made by 50 G oral glucose challenge test (value RBS ≥140mg/dl), confirmation being done by OGTT.^11^ Non-probability purposive sampling was done and after written informed consent, 25 healthy females with no co-morbidity as control (**Group A)** and 62 GDM females were enrolled in study. Thirty of these GDM patients, with blood sugar levels less than 130 mg/dl were advised life style modification, including exercise and diet control, were kept in **Group B**. They were advised to take only 2500 calories per day with provision of suitable diet charts and 30 minutes of walk thrice weekly. Thirty-two GDM patients in **Group C** were with RBS more than 130 mg/dl received tablet Metformin 500mg along with strict diet control therapy (<2500 calories/ day and 30 minutes of walk thrice weekly). Metformin was started with 500 mg then was increased up to 1500 mg according to the tolerance and glycemic values of the patient. These patients were followed regularly in the antenatal clinics till term. On every visit their treatment, any side effects and blood sugar level were assessed and proper management was reassured. Patients (5 in group B, 7 in group C) were excluded from the study as they delivered elsewhere or were added insulin for the treatment with Metformin. Placentae were collected within 30-40 minutes of delivery from these patients and were preserved in 10% formalin in labeled containers. Gross morphological evaluation of placenta was done (Table-I & II), following the dissection and tissues were taken from 6 o’clock, 12o’clock and central areas for slide preparation. The sections were treated with alcohol and xylene and then blocks were prepared using paraffin wax. For slide preparation, sections were sliced into 4mm thin films with manual microtome and slides were prepared. These slides were finally stained with hematoxyline and eosin, PAS and trichome stains and after drying, visualized under light microscope. Microscopically placental hypoxic parameters were observed. (Table-III).^12^

**Table-I:** Gross Morphology within and between groups N=75 (n=25).

S. No	Groups	Characteristics						

		Placental size1(cm)	Placental size2 (cm)	Placental width (cm)	Placental weight (gm)	Cord length (cm)	Cord width (cm)	Cord vessels (n)
1	Group A	16.32±2.34	14.00±1.91	2.12±0.58	567.6±138.9	41.98±7.88	1.34±0.45	3±0
2	Group B	15.06±2.41	12.88±2.92	2.84±0.62	590±147.9	42.96±7.4	1.84±0.34	3±0
3	Group C	15.88±2.58	13.92±2.72	2.20±0.5	626.4±122.6	45.54±7.37	1.68±0.45	3±0

*P-value*								

1	Group B v/s A	0.06	0.12	0.00*	0.58	0.65	0.00*	NA
2	Group C v/s A	0.5	0.9	0.6	0.119	0.1	0.01*	NA
3	Group B v/s C	0.25	0.12	0.001*	0.34	0.22	0.16	NA

Key:Group A: Normal control group, Group B: GDM females on diet control, Group C: Metformin treated group,

* Statistically significant, NA: Independent t test not applicable.

**Table-II:** Gross morphology within and between groups N=75(n=25).

S.No	Groups	Characteristics													
		Placental consistency		Placental shape		Color of membrane		Cord insertion		Cord knots		Cord strictures		Cord hematoma	
		Soft	Hard	Discoid	Other	Blue	Pale	Central	Peripheral	P	A	P	A	P	A
1	Group A	17(68%)	8(32%)	19(76%)	6(24%)	8(32%)	17(68%)	7(28%)	18(72%)	2(8%)	23(92%)	1(4%)	24(96%)	9(36%)	16(64%)
2	Group B	16(64%)	9(36%)	19(76%)	6(24%)	11(44%)	14(56%)	8(32%)	17(68%)	6(24%)	19(76%)	6(24%)	19(76%)	5(20%)	20(80%)
3	Group C	16(64%)	9(36%)	17(68%)	8(32%)	11(44%)	14(56%)	10(40%)	15(60%)	3(12%)	22(88%)	6(24%)	19(76%)	10(40%)	15(16%)
*P-value*															
4	Group B v/s A	0.76		>0.99		0.38		0.75		0.24		0.09^		0.2	
5	Group C v/s A	0.76		0.52		0.38		0.37		>0.99		0.09^		0.77	
6	Group B v/s C	>0.99		0.52		>0.99		0.5		0.46		>0.99		0.12	

**Table-III:** Microscopic morphology within and between groups N=75 (n=25).

S. No	Groups	Characteristics											

		Villous immaturity		Chorangiosis		Infarction		Villous fibroid necrosis		Calcification		Syncytial Knots	

		P	A	P	A	P	A	P	A	P	A	P	A
1	Group A	4(16%)	21(64%)	6(24%)	19(76%)	7(28%)	18(72%)	18(72%)	7(28%)	11(44%)	14(56%)	4(16%)	21(84%)
2	Group B	10(40%)	15(60%)	13(52%)	12(48%)	14(56%)	11(44%)	19(76%)	6(24%)	10(40%)	15(60%)	14(56%)	11(44%)
3	Group C	5(20%)	20(805)	5(20%)	20(80%)	9(36%)	16(64%)	22(88%)	3(12%)	15(60%)	10(40%)	7(28%)	18(72%)

*P-value*													

4	Group B v/s A	0.05*		0.04*		0.04*		0.74		0.77		0.003*	
5	Group C v/s A	>0.9^		0.73		0.54		0.15		0.25		0.3	
6	Group B v/s C	0.12		0.01*		0.15		0.24^		0.15		0.04*	

Key:Group A: Normal control group, Group B: GDM females on diet control, Group C: Metformin treated group,

* Statistically significant, P: Present, A: Absent,

^ fisher exact test applied due to decrease cell count.

Data was tabulated and analyzed by SPSS version 16. P value of 0.05 or less was considered statistically significant for the results. For the numerical variables mean with Independent t test and for categorical variables percentages with chi square tests were applied for evaluation. Fisher exact test was used where chi square test was not applicable due to decreased cell counts.

## RESULTS

Significant differences were observed in FBS, RBS, HbA_I_C as they were diabetics and non-diabetics. HbA_I_C evaluated at 36 weeks of pregnancy has shown significant decrease in group C as compared to group B. (Table-IV)

**Table-IV:** Maternal characteristics group (B v/s A) (C v/s A) & (B v/s C) N=75.

S. No.	Maternal Characteristics	AGE (years)	WEIGHT (kg)	FBS (mg/dl)	RBS (mg/dl)	HbA_I_C 1	HbA_I_C 2

		Mean ± S.D	Mean ± S.D	Mean ± S.D	Mean ± S.D	%	%
1	Group A	29.0±4.37	73.84±9.97	72.24±9.34	126.8±35.8	4.84	4.97
2	Group B	30.08±3.16	78.54±6.93	90.9±16.8	148.72±38.9	5.34	5.74
3	Group C	29.76±3.41	77.9±7.6	104.4±13.12	171±37.44	5.28	5.42

*P-value*							

1	Group B v/s A	0.32	0.059	0.00*	0.03*	0.00*	0.00*
2	Group C v/s A	0.49	0.11	0.00*	0.00*	0.001*	0.00*
3	Group B v/s C	0.75	0.75	0.00*	0.04*	0.693	0.01*

Group A: Normal control group, Group B: GDM females on diet control, Group C: Metformin treated group,

* Statistically significant, FBS: fasting blood sugar at the time of enrollment;RBS: Random blood sugar at the time of enrollment, HbA_I_C 1: at the time of patient enrollment, HbA_I_C 2: at 36 weeks of gestation.

Results were statistically significant between group B and A for placental width and cord width (p value of 0.00). Comparison of group C and A showed only significant results for cord width (p=0.01), while other parameters were similar. The results were statistically significant for placental width when group B and C were compared (p= 0.001) and all remaining parameters were non-significant, showing that diet controlled GDM placentae were thicker than Metformin treated GDM placentae. (Table-I)

The results between all the three groups (normal control, GDM on diet therapy, GDM on metformin therapy) were statistically non-significant. (Table-II)

On light microscopy hypoxic placental features between group B and A were statistically significant for villous immaturity, chorangiosis, infarction and syncytial knots (p=0.05, 0.04, 0.04 and 0.03 respectively). (Fig.1). For group C and A, all the microscopic parameters were statistically non-significant, showing that metformin treated placentae were similar to normal controlled placentae. (Fig.1). For group B and C for microscopic parameters, results were significant for chorangiosis and syncytial knots formation (p= 0.01 & 0.04 respectively) showing that the diet controlled placentae had chorangiosis and syncytial knots when compared to Metformin treated placentae. (Table-III)

**Fig.1A:**
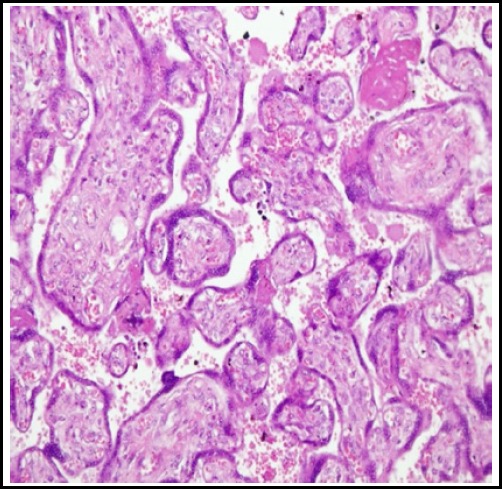
Histology of normal healthy placenta.

**Fig.1B:**
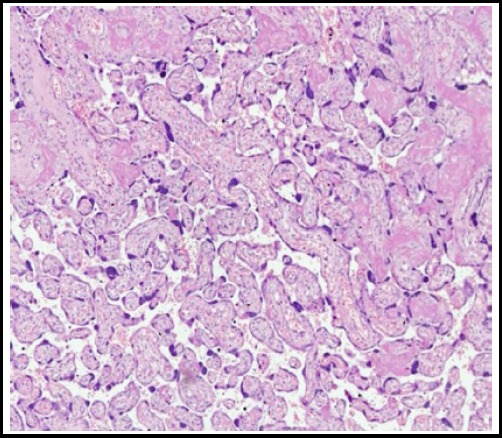
Diet control placenta.

**Fig.1C:**
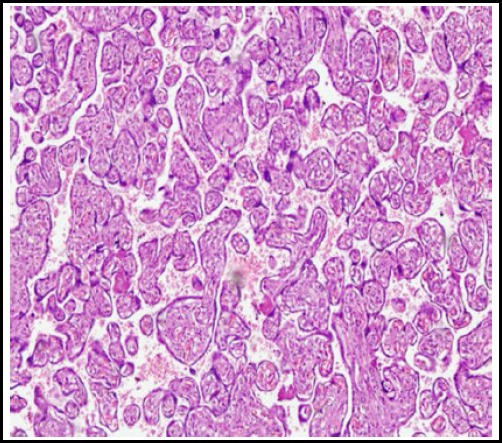
Metformin treated placentae.

## DISCUSSION

GDM is associated with structural and functional alterations in placenta leading to fetal hypoxia, fetal morbidity and still birth^13^. These can be envisioned by gross and microscopic changes in the morphology of placentae, the main communicating tissue between mother and the growing fetus.

Changes in placental morphology due to hypoxia in GDM include immaturity of villi, ischemia, villious fibrotic and necrotic patches. Chorangiosis (presence of more than 5 vessels per villi), calcification, syncytial knots formation (excessive amounts and formation of trophoblastic nuclear out pouching) are also important signs of altered placental texture.^14^ These increased hypoxic parameters in placental tissues are associated with increased in fetal and maternal mortality and morbidity. Proper diagnosis and management significantly reduces the risks for complications.^15^ In this study two groups of GDM, one on diet therapy and other on Metformin treatment were compared with each other and with the normal control.

It was expected that placentae of diet controlled patients would not differ much from normal morphology because the glucose levels were strictly kept near to normal, but significant change in placental gross morphology of GDM patients were observed despite strict diet control. Leo et al. 1977; documented that the placentae of diet controlled patients are slightly heavier in weight than normal placentae^16^ and was similar to our findings (590±147.9gms vs 567.6±138.9gms). Kucuk et al. also indicated that GDM patients kept on diet only has increased placental weight as compared to normal. (694.8±152.1 gms v/s 610.2±116.6gms).^17^ Our study also indicated that the placental width and cord width were significantly more in these diet treated GDM patients. The reason could be that diet alone is unable to oppose the altered levels of placental vascular endothelial growth factor, insulin and other growth factors (IGF1, IGF2 and IGF binding proteins) which regulate the placental development and are altered in GDM leading to thickened placenta and its cord.^18^

Diet controlled placentae were significantly different from normal placentae as they showed villous immaturity, infarction, chorangiosis and syncytial knots formation when compared to normal control. Verma et al. 2010 has documented that in GDM treated with diet only, placentae showed fibrosis and ischemic changes, more syncytial knots, mild edema and fibrinoid necrosis, which is similar to our results.^19^ Placentae treated with Metformin revealed non-significant results when compared to normal control in gross placental morphology except for cord width. All remaining gross and microscopic hypoxic parameters were non-significant between the groups, making it near to control. Metformin treated placenta had significantly less thickness, chorangiosis and syncytial knot formation as compared to diet controlled placentae when light microscopic results were evaluated. Remaining microscopic hypoxic parameters were also numerically lowered in metformin group than diet control group.

Using multiple related key words and utilizing search such as engines google.com, Google scholar, PubMed, Science direct, Wiley.com from 1980 till Dec 2015, no documentation was found on the details of placental morphology with Metformin in GDM. Except a single case documented by Campbell in 2009 in which a GDM patient with preeclampsia on Metformin had intrauterine death and the placental morphology showed pronounced changes such as villous dysmaturity, chorioamniotis, villi fibrosis.^20^ However it was not clarified that the placental changes were purely because of gestational diabetes or the combination of GDM with hypertension had produced them.

Most of the in-vivo and in-vitro studies have shown that Metformin produces its anti-diabetic effect by decreasing the gluconeogenesis primarily through the inhibition of lactate uptake in adipocytes. Other main mechanism reported is decrease in ATP concentration in hepatocytes, leading to decrease in glucose production from glycogen in hepatocytes. At cellular level it also disrupts the respiratory chain oxidation in mitochondria of the liver cell.^21^ Also, Metformin significantly lower down the HbA_I_C which could add on in its beneficial effects in GDM patients. These multiple actions of Metformin in diabetic cells could be the reason of its beneficial effects on the placenta as compared to diet control only.

In conclusion, when we compared the placental morphology of diet controlled and Metformin treated placentae to the normal control placentae, Metformin treated placentae had morphology near to normal placentae whereas diet control had shown significant gross and histological changes. Large sample sized studies of Metformin treated placenta, based on electron microscopy and immuno-histochemistry testing would be an open avenue for the new researchers.

## CONCLUSION

In GDM patients, Metformin and diet control produced beneficial effects on placental morphology being comparable to normal control and in contrast to diet plus exercise groups.

## References

[1] Territi K, Ekbald U, Vehlberg T, Ronnemaa T (2008). Comparison of metformin and insulin in the treatment of gestational diabetes: A retrospective;case control study. Rev Diabet Stud.

[2] Ben Haroush A, Yogev V, Hod M (2003). Epidemiology of gestational diabetes mellitus and its association with Type 2 diabetes. Diabetic Med.

[3] Hiden U, Glitzner E, Hartmann M, Desove G (2009). Insulin and IGF system in human placenta of normal and diabetic pregnancies. J Anat.

[4] Cowet RM (2002). The infant of diabetic mother. Neo Rev.

[5] Janson T, Certin I, Powell TL, Desoye G, Radaelli T, Ericsson A (2006). Placental transport and metabolism in fetal overgrowth, a workshop report. Placenta.

[6] Akhter F, Anjuman Bano ML, Ferdaus R (2010). Effects of gestational diabetes mellitus on gross morphological structure of preterm placenta. Bangladesh J Anat.

[7] Tewari V, Tewari A, Bhardwaj N (2011). Histological and histo-chemical changes in placenta of diabetic pregnant females and its comparison with normal placenta. Asian Pacific J Tropical Dis.

[8] Gutzin SJ, Kozer E, Magee LA, Feig DS, Koren G (2003). The safety of oral hypoglycemic agents in first trimester of pregnancy: A meta-analysis. Can J Clini Pharm.

[9] Singh AK, Singh R (2015). Metformin in gestational diabetes: An emerging contender, Indian. J Endocr Metab.

[10] Gandhi P, Bustani R, Madhuvrata P, Farrell T (2012). Introduction of metformin for Gestational diabetes mellitus in clinical practice: Has it had an impact. Euro J Obstet Gynae Rep Bio.

[11] Perkins JM, Dunn JP, Jagasia SM (2007). Perspectives in Gestational Diabetes Mellitus;A review of screening, Diagnosis and Treatment. Clin Diabetics.

[12] Benirschke K, Lewis SH, Stacey E Placenta. Histology of pathologists.

[13] Madazal R, Tuten A, Calary Z, Uzun H, Uludag S, Ocak V (2008). Incidence of placental abnormalities, maternal and cord plasma malondialdehyde and vascular endothelial growth factors levels in women with gestational diabetes mellitus and non-diabetic controls. Gynecol Obstet Invest.

[14] Ghidin A, Salafia CM (2005). Histological placental lesions in women with recurrent preterm delivery. Acta Obstet Gynacol Scand.

[15] Spaight C, Gross J, Horch A, Pudder JJ (2016). Gestational Diabetes Mellitus. Endocr Dev.

[16] Leo TT, Lee CP, Wong WM (1997). Placental weight to birth weight ratio is increased in mild gestational glucose intolerance. Placenta.

[17] Kuck M, Doymaz F (2009). Placental weight and placental weight-to-birth weight ratio are increased in diet- and exercise-treated gestational diabetes mellitus subjects but not in subjects with one abnormal value on 100-g oral glucose tolerance test, Journal of Diabetes and its Complications. J Diabetes Complications.

[18] Hiden U, Glitzner E, Hartmann M, Desove G (2009). Insulin and IGF system in human placenta of normal and diabetic pregnancies. J Anat.

[19] Verma R, Mishra S, Kaul JM (2010). Cellular changes in the placenta in pregnancies complicated with diabetes. Int J Morphol.

[20] Cambell IW, Duncan C, Urquhart R, Evans M (2009). Placental dysfunction and still birth in gestational diabetes mellitus. Br J Diabetes Vascular Dis.

[21] Kirpichnikov D, Macferlarne SL, Sowers JR (2002). Metformin: An update. Ann Intern Med.

